# Supplementary material for: ANCA Associated Glomerulonephritis Following SARS-CoV-2 Vaccination: A Case Series and Systematic Review
Source: Vaccines (Basel). 2023 May 15;11(5):983. doi: 10.3390/vaccines11050983 (PMC10223042; doi:10.3390/vaccines11050983)
Supplement: Supplementary file 1 [file vaccines-11-00983-s001.zip › vaccines-2328504-supplementary.pdf]

vaccines

Systematic Review

ANCA associated glomerulonephritis following SARs-CoV2 vaccination: A Case Series and Systematic Review

Supplementary Material

Table S1. Clinical spectrums and outcomes of ANCA-associated glomerulonephritis following SARS-CoV-2 vaccination**.**

No	Study	Age	G	Vaccine types	Dose	Onset	Symptoms	Extra-renal	ANCA (IF)	ANCA Ab	AutoAb	Previous sCr (mg/dL)	Peak sCr (mg/dL)	Kidney pathology	D/R	Treatment	Outcome
1	Anderegg, 2021 [1]	81	M	Moderna	2nd	Shortly	AKI	Bilateral necrotic mass of lungs	N/A	PR3	N/A	N/A	N/A	cGN, FN, vasculitis	D	GC, CY, PEX	Remission
2	Chen, 2021 [2]	70	F	Moderna	1st	3 wk	Hematuria, hemoptysis, AKI	Bilateral peribronchial consolidation	N/A	MPO	N/A	1	3.5	cGN, vasculitis	D	GC, RTX, PEX	Remission
3	Davidovic, 2021 [3]	54	F	Pfizer	2nd	2 wk	Episcleritis, constitutional symptoms	Eye involvement, discrete opacities in both lungs	N/A	MPO	N/A	N/A	2.1	cGN (7/21)	D	GC, RTX	Remission
4	Dube, 2021 [4]	29	F	Pfizer	2nd	16 d	AKI, Nephritic	No	N/A	MPO	RF	0.8	1.9	cGN (9/44), fcGN (5/44), fGN (7/44), GS 8/44), mild AIN	D	GC, CY	Remission
5	Feghali, 2021 [5]	58	M	Moderna	2nd	4 d	N/V, weight loss	Right upper lobe consolidation, bilateral pleural effusion	cANCA	PR3	Neg	normal	4.1	diffuse cGN, segmental FN	D	GC, CY, RTX, PEX	Remission
6	Hakroush, 2021 [6]	79	F	Pfizer	2nd	2 wk	Weakness, thigh pain	Rhabdomyolysis	Positive	MPO	ANA 1:360	0.7	6.6	cGN (1/15), GS (2/15) , eosinophilic AIN, myoglobin casts, ATN	D	GC, CY	Remission
7	Schaubschlager, 2021 [7]	77	F	Moderna	2nd	12 wk	AKI	No	N/A	MPO	ANA 1:80	0.9	2.9	cGN and fcGN (11/22), fGN (1/22), GS (4/22), FN	D	GC, RTX	Remission
8	Sekar, 2021 [8]	52	M	Moderna	2nd	2 wk	Headache, weakness	No	cANCA	PR3	Neg	1.1	8.4	cGN (38/46), FN, ATN	D	GC, RTX, CY, HD	Dialysis
9	Shakoor, 2021 [9]	78	F	Pfizer	1st/ 2nd	16 d/ 12 d	N/V, diarrhea	No	N/A	MPO	N/A	0.8	1.3/ 3.5	No biopsy/ cGN, FN, moderate AIN	D/R	No treatment/GC, RTX	Remission with relapse
10	Villa, 2021 [10]	63	M `	AstraZeneca	1st	7 d	Hemoptysis, RPGN	Lung	pANCA	MPO	Neg	normal	2.9	cGN	D	GC, CY	Remission
11	David, 2022 [11]	75	M	AstraZeneca	1st	5 wk	Hemoptysis	Pulmonary hemorrhage	pAnCA	MPO	N/A	2.6	7	cGN	R	GC, RTX, HD	Dialysis
12		74	M	AstraZeneca	2nd	2 wk	AKI	No	pANCA	MPO	N/A	0.9	10	cGN with vasculitis	D	GC, CY, HD	Remission
13	Garcia, 2022 [12]	78	F	CoronaVac	2nd	26 d	Constitutional symptoms, cough, AKI	No	cANCA	PR3	ANA, RF	0.8	7.3	cGN (6/14), necroticing GN (9/14), FN, ATN	D	GC, CY, HD, MMF	Remission
14	El hasbani, 2022 [13]	47	F	Pfizer	1st	3 d	Flank pain, weakness, edema	No	N/A	MPO	N/A	0.8	2.9	fGN, IF/TA	D	GC, azathioprine	Remission
15	Kim, 2022 [14]	72	F	2AstraZeneca, Moderna	3rd	18 d	Constitutional symptoms, AKI	Otologic symptoms, gastritis	Positive	MPO	RF 221 IU/mL	0.8	4.7	fcGN (42.9%), GS (14.3%), AIN	D	GC, CY	Remission
16	Loo, 2022 [15]	75	F	Moderna	1st	11 wk	AKI	No	N/A	MPO	N/A	N/A	6.3	cGN	D	GC, RTX, HD	Remission

17	Ma, 2022 [16]	70	F	CoronaVac	1st	10 d	Constitutional symptoms, foamy urine	No	pANCA	MPO	ANA	0.5	5.4	cGN (4/34), fcGN (10/34), fGN (3/34),	D	GC, CY	Remission
18	Noel, 2022 [17]	62	F	Pfizer	2nd	4 wk	AKI	No	pANCA	MPO	ANA 1:160 speckled	1.0	5.2	cGN (5/57), FN, severe IF/TA	D	GC, CY, MMF	Remission
19	Obata, 2022 [18]	84	M	Moderna, Pfizer	2nd	2 wk	Constitutional symptoms, cough	Interstitial pneumonia	N/A	MPO	N/A	1.2	1.2	cGN (6/20), GS (3/20), FN	D	GC	Remission
20	Prabhahar, 2022 [19]	51	M	AstraZeneca	1st	15 d	AKI, fever, polyarthritis	Arthritis	N/A	PR3	Neg	1.2	4.8	cGN (19/20)	D	GC, RTX	Remission
21	Ramezanzade, 2022 [20]	15	M	Sinopharm	2nd	1 mo	Constitutional symptoms, N/V, flank pain	No	pANCA	MPO	N/A	N/A	3	cGN, necrotizing GN, mild AIN, ATN	D	GC, MMF	Remission without relapse
22	So, 2022 [21]	42	M	Pfizer	2nd	63 d (after 1st dose)	Gross hematuria edema, constitutional symptoms	No	N/A	MPO	Neg	1.0	3.1	cGN (9/21), fcGN (2/21), mild interstitial infiltration	D	GC, RTX, PEX	Remission
23	Suzuki, 2022 [22]	72	M	Pfizer	2nd	1 d	Constitutional symptoms, AKI	No	N/A	MPO	ANA 160 IU/mL,	normal	7.4	cGN, segmental necrosis, FN in small vessels	D	GC, RTX, HD	Remission
24	Yadav, 2022 [23]	52	F	Johnson and Johnson	1st	12 d	Fever, joint pain, weakness	Arthralgia	pANCA/cANCA	N/A	N/A	N/A	6.1	cGN	D	GC, CY	N/A
25	Zamoner, 2022 [24]	58	F	AstraZeneca	1st	5 d	Constitutional symptoms, arthralgia, foamy urine, HT	No	N/A	MPO	Neg	N/A	2.2	cGN, fcGN, fGN	D	GC, CY, azathioprin	Remission
26	Bansal, 2023 [25]	67	F	BBV152/Covaxin	2nd	2 wk	Edema, N/V decrease appetite	No	pANCA	MPO	Neg	1.2	6.4	cGN (3/6), 1fcGN(1/6), fGN (1/6), FN	D	GC, CY, HD	Remission
27		76	F	AstraZeneca	2nd	20 d	Fever, weight loss, productive cough	Usual interstitial pneumonia	pANCA	MPO (10.61 RU/mL)	ANA 1:1280 speckled, RF 29.6 IU/mL	1.5	3.5	cGN (7/24), GC (7/24)	D	GC, CY, HD	Dialysis
28	Our study, 2023	69	F	Pfizer	3rd	4 wk	Constitutional symptoms, hearing loss, AKI	Sensorineural hearing loss	pANCA	MPO	ANA 1:80 speckled, DCT 1+, pos cryoglobulin	0.7	7.1	cGN (10/13), GS (2/13), eosinophil AIN, FN, vasculitis	D	GC, CY, PEX	Remission
29		84	F	Moderna	3rd	1 mo	Constitutional symptoms, hearing loss, AKI	Wallerian degeneration, optic neuritis	cANCA	MPO	DCT 1+	0.9	5.1	cGN (7/20), GS (2/20), FN, eosinophilic AIN	D	GC, IVIG, HD	Remission

Abbreviations: AIN, acute interstitial nephritis; ANCA, antineutrophil cytoplasm antibody; anti-GBM, anti-glomerular basement membrane; ANA, Antinuclear antibodies; ATN, acute tubular necrosis; cANCA, cytoplasmic ANCA; cGN, crescentic glomerulonephritis; CY, cyclophosphamide; D, *De novo* GN; fcGN, fibrocellular crescentic glomerulonephritis; fGN, fibrous crescent glomerulonephritis; FN, fibrinoid necrosis; F, female; G, gender; GS, glucocorticoids; glomerulosclerosis; HD, hemodialysis; IVIG, intravenous immunoglobulin; IF/TA, interstitial fibrosis with tubular atrophy; M, male; MPO, anti-myeloperoxidase antibody; N/V nausea/vomiting; pANCA, perinuclear ANCA; PR3, anti-proteinase 3; R, relapsed GN; RF, rheumatoid factor; RTX, rituximab; sCr, serum creatinine.

Supplementary References

1. Anderegg, M.A.; Liu, M.; Saganas, C.; Montani, M.; Vogt, B.; Huynh-Do, U.; Fuster, D.G. De novo vasculitis after mRNA-1273 (Moderna) vaccination. *Kidney International* **2021**, *100*, 474-476, doi:10.1016/j.kint.2021.05.016.
2. Chen, C.C.; Chen, H.Y.; Lu, C.C.; Lin, S.H. Case Report: Anti-neutrophil Cytoplasmic Antibody-Associated Vasculitis With Acute Renal Failure and Pulmonary Hemorrhage May Occur After COVID-19 Vaccination. *Frontiers in Medicine* **2021**, *8*, doi:10.3389/fmed.2021.765447.
3. Davidovic, T.; Schimpf, J.; Sprenger-Mähr, H.; Abbassi-Nik, A.; Soleiman, A.; Zitt, E.; Lhotta, K. De Novo and Relapsing Glomerulonephritis following SARS-CoV-2 mRNA Vaccination in Microscopic Polyangiitis. *Case Reports in Nephrology* **2021**, *2021*, doi:10.1155/2021/8400842.
4. Dube, G.K.; Benvenuto, L.J.; Batal, I. Antineutrophil Cytoplasmic Autoantibody–Associated Glomerulonephritis Following the Pfizer-BioNTech COVID-19 Vaccine. *Kidney International Reports* **2021**, *6*, 3087-3089, doi:10.1016/j.ekir.2021.08.012.
5. Feghali, E.J.; Zafar, M.; Abid, S.; Santoriello, D.; Mehta, S. De-novo Antineutrophil Cytoplasmic Antibody-Associated Vasculitis Following the mRNA-1273 (Moderna) Vaccine for COVID-19. *Cureus* **2021**, *13*, e19616, doi:10.7759/cureus.19616.
6. Hakroush, S.; Tampe, B. Case Report: ANCA-Associated Vasculitis Presenting With Rhabdomyolysis and Pauci-Immune Crescentic Glomerulonephritis After Pfizer-BioNTech COVID-19 mRNA Vaccination. *Frontiers in Immunology* **2021**, *12*, doi:10.3389/fimmu.2021.762006.
7. Schaubschlager, T.; Rajora, N.; Diep, S.; Kirtek, T.; Cai, Q.; Hendricks, A.R.; Shastri, S.; Zhou, X.J.; Saxena, R. De novo or recurrent glomerulonephritis and acute tubulointerstitial nephritis after COVID-19 vaccination: A report of six cases from a single center. *Clinical Nephrology* **2022**, *97*, 289-297, doi:10.5414/CN110794.
8. Sekar, A.; Campbell, R.; Tabbara, J.; Rastogi, P. ANCA glomerulonephritis after the Moderna COVID-19 vaccination. *Kidney International* **2021**, *100*, 473-474, doi:10.1016/j.kint.2021.05.017.
9. Shakoor, M.T.; Birkenbach, M.P.; Lynch, M. ANCA-Associated Vasculitis Following Pfizer-BioNTech COVID-19 Vaccine. *American Journal of Kidney Diseases* **2021**, *78*, 611-613, doi:10.1053/j.ajkd.2021.06.016.
10. Villa, M.; Díaz-Crespo, F.; Pérez de José, A.; Verdalles, Ú.; Verde, E.; Almeida Ruiz, F.; Acosta, A.; Mijaylova, A.; Goicoechea, M. A case of ANCA-associated vasculitis after AZD1222 (Oxford-AstraZeneca) SARS-CoV-2 vaccination: casualty or causality? *Kidney Int* **2021**, *100*, 937-938, doi:10.1016/j.kint.2021.07.026.
11. David, R.; Hanna, P.; Lee, K.; Ritchie, A. Relapsed ANCA associated vasculitis following Oxford AstraZeneca ChAdOx1-S COVID-19 vaccination: A case series of two patients. *Nephrology* **2022**, *27*, 109-110, doi:10.1111/nep.13993.
12. Garcia, D.S.; Martins, C.; da Fonseca, E.O.; de Carvalho, V.C.P.; de Rezende, R.P.V. Clinical Images: Severe proteinase 3 antineutrophil cytoplasmic antibody glomerulonephritis temporally associated with Sinovac Biotech's inactivated SARS-CoV-2 vaccine. *ACR Open Rheumatology* **2022**, *4*, 277-278, doi:10.1002/acr2.11397.
13. El Hasbani, G.; Uthman, I. ANCA-Associated Vasculitis following the First Dose of Pfizer-BioNTech COVID-19 Vaccine. *Nephron* **2022**, 1-5, doi:10.1159/000525562.
14. Kim, B.C.; Kim, H.S.; Han, K.H.; Han, S.Y.; Jo, H.A. A Case Report of MPO-ANCA-Associated Vasculitis Following Heterologous mRNA1273 COVID-19 Booster Vaccination. *Journal of Korean Medical Science* **2022**, *37*, doi:10.3346/jkms.2022.37.e204.
15. Loo, H.T.; Hsu, C.H.; Chen, L.F. HLA-DR4 and DRB4: Potential risk alleles for COVID-19 vaccination-related ANCA-associated vasculitis. *Therapeutic Apheresis and Dialysis* **2022**, doi:10.1111/1744-9987.13925.
16. Ma, Y.; Huang, T.; Xu, G. ANCA-associated vasculitis following the CoronaVac vaccination. *Therapeutic Advances in Chronic Disease* **2022**, *13*, doi:10.1177/20406223221125708.
17. Noel, E.; Rashid, U.; Rabbani, R.; Khan, W.A.; Benjamin, Y.S.; Lee, I. Antineutrophil Cytoplasmic Autoantibody-Associated Glomerulonephritis as a Possible Side Effect of COVID-19 Vaccination. *Cureus* **2022**, *14*, e30565, doi:10.7759/cureus.30565.

18. Obata, S.; Hidaka, S.; Yamano, M.; Yanai, M.; Ishioka, K.; Kobayashi, S. MPO-ANCA-associated vasculitis after the Pfizer/BioNTech SARS-CoV-2 vaccination. *Clinical Kidney Journal* **2022**, *15*, 357-359, doi:10.1093/ckj/sfab181.
19. Prabhahar, A.; Naidu, G.S.R.S.N.K.; Chauhan, P.; Sekar, A.; Sharma, A.; Sharma, A.; Kumar, A.; Nada, R.; Rathi, M.; Kohli, H.S.; et al. ANCA-associated vasculitis following ChAdOx1 nCoV19 vaccination: case-based review. *Rheumatology International* **2022**, *42*, 749-758, doi:10.1007/s00296-021-05069-x.
20. Ramezanzade, E.; Ghanbari, R.; Yazdanipour, T. Antineutrophil Cytoplasmic Antibody (ANCA)-Associated Glomerulonephritis in a 15-year-old Patient After Receiving the Second Dose of the BBIBP-CorV (Sinopharm) COVID-19 Vaccine: A Case Report. *Nephro-Urology Monthly* **2022**, *14*, doi:10.5812/numonthly-127124.
21. So, D.; Min, K.W.; Jung, W.Y.; Han, S.W.; Yu, M.Y. Microscopic Polyangiitis Following mRNA COVID-19 Vaccination: A Case Report. *Journal of Korean Medical Science* **2022**, *37*, doi:10.3346/jkms.2022.37.e154.
22. Suzuki, M.; Sekiguchi, Y.; Sasaki, M.; Inaba, S.; Oyama, S.; Inoue, Y.; Warabi, M.; Ohashi, K.; Inoshita, S. Antineutrophil Cytoplasmic Antibody-associated Vasculitis after COVID-19 Vaccination with Pfizer-BioNTech. *Internal Medicine* **2022**, *61*, 2925-2929, doi:10.2169/internalmedicine.9807-22.
23. Yadav, R.; Shah, S.; Chhetri, S. ANCA-associated vasculitis following Johnson and Johnson COVID-19 vaccine. *Annals of Medicine and Surgery* **2022**, *79*, doi:10.1016/j.amsu.2022.104123.
24. Zamoner, W.; Scardini, J.B.; De Dio, B.J.; Marques, A.D.M.; Silva, V.D.S.; Garcia, A.L.; dos Santos, D.C.; Viero, R.M. ANCA-associated vasculitis following Oxford-AstraZeneca COVID-19 vaccine in Brazil: Is there a causal relationship? A case report. *Frontiers in Medicine* **2022**, *9*, doi:10.3389/fmed.2022.1003332.
25. Bansal, S.B.; Rana, A.S.; Manhas, N.; Rana, A. Post COVID Vaccination (COVAXIN™ -BB152 V) Pauci-immune Crescentic Glomerulonephritis. *Indian J Nephrol* **2022**, *32*, 495-497, doi:10.4103/ijn.ijn_352_21.
